# A child with a foreign body in bronchus misdiagnosed as asthma

**DOI:** 10.1002/ccr3.3153

**Published:** 2020-07-21

**Authors:** Nagendra Chaudhary, Sandeep Shrestha, Om P. Kurmi

**Affiliations:** ^1^ Department of Pediatrics Universal College of Medical Sciences Bhairahawa Nepal; ^2^ Department of Medicine Population Health Research Institute McMaster University Hamilton ON Canada

**Keywords:** bronchial asthma, bronchus, children, foreign body aspiration, misdiagnosis

## Abstract

Foreign body ingestion should be considered as an important differential in a child with difficult asthma. We report an 11‐year‐old male child with foreign body aspiration who initially was diagnosed and treated as difficult asthma. Later on, he was diagnosed to have a foreign body in the right bronchus, which was successfully removed by flexible bronchoscopy.

## Graphical abstract

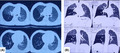

## 1 INTRODUCTION

Foreign body (FB) aspiration is common in infants and younger children with a high incidence in toddlers as they use their mouth to explore the surroundings. It can lead to high mortality and morbidity if it remains undiagnosed and unaddressed appropriately in time.^1^, ^2^ The most common types of FB aspiration in children are seeds, peanuts, food particles, and toys. Items such as coins, paper clips, pins, and pen caps are usually noticed to be aspirated in older children.^3^, ^4^

The diagnosis of FB aspiration in airways is difficult in cases with an uncharacterized medical history and discrete symptoms. Delay in the diagnosis of a FB is mainly due to parents not witnessing the choking crisis along with failure to diagnose the condition by the primary physician because of its atypical presentation and misleading radiological findings, which often leads to therapeutic challenges.^5^

Aspiration of organic FB leads to airway mucosal inflammation and edema in the acute stage. Further, if FB in airways is missed, granulation tissue forms, which later on can lead to symptoms masquerading difficult bronchial asthma along with recurrent/persistent pneumonia.

Here, we report an 11‐year‐old child with FB aspiration who initially was misdiagnosed and treated as asthmatic patient. Later on, he was diagnosed to have FB in the right bronchus, which was successfully removed by flexible bronchoscopy.

## 2 CASE REPORT

An 11‐year‐old boy was brought to the pediatric outpatient department with complaints of recurrent cough, tightness of chest, and breathing difficulty for the last 18 months. The child was a product of nonconsanguineous parents. The child was asymptomatic 18 months prior and doing well. Developmentally, he was normal, and his scholastic performances were normal. There was no reported family history of asthma or tuberculosis.

**FIGURE 1:**
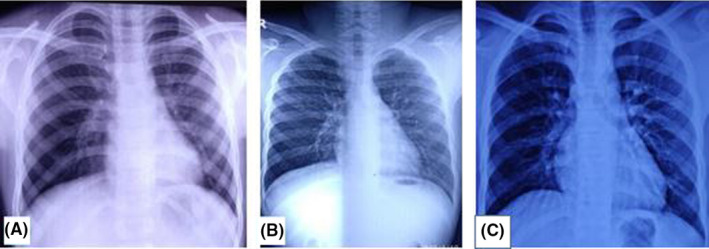
Chest X‐ray showing (A) hyperinflated right lung field; (B) hyperinflated right lung field with right middle and lower lobes infiltrates; (C) normal lung fields following removal of FB

Eighteen months back, the child developed sudden‐onset cough, wheeze, and fever for which he was diagnosed as pneumonia and treated with oral antibiotics (amoxicillin‐clavulanic acid) and bronchodilators (oral salbutamol syrup; nebulization—salbutamol, ipratropium bromide, and budesonide) by his local pediatrician. Chest X‐ray showed right‐sided hyperinflation (Figure 1A). The child responded well to the above treatment. Later on, the child had repeated reoccurrence of symptoms for which he received several courses of oral antibiotics (amoxicillin‐clavulanic acid) along with bronchodilators (oral salbutamol syrup) and nebulization (salbutamol, ipratropium bromide, and budesonide).

**FIGURE 2:**
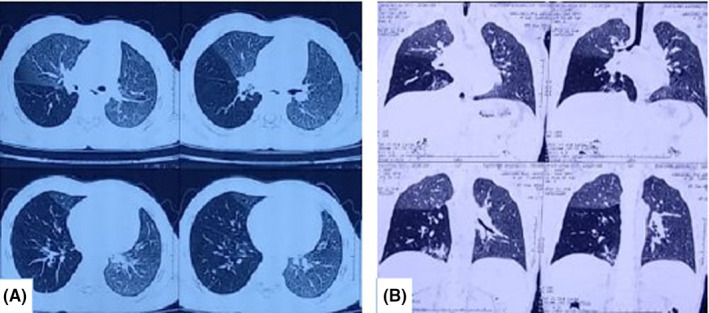
A and B, Showing patchy areas of consolidation involving anterior basal segment of right lower lobe with air bronchograms within it with evidence of short segment partial cutoff of right lower lobe bronchus with suggestion of ill‐defined intraluminal soft tissue

The child visited our center after one year of the onset of symptoms with frequent similar episodes of cough and breathing difficulty (weekly night‐time symptoms). The anthropometric examination was appropriate for his age. On chest examination, there were diffuse rhonchi heard. Blood investigation showed hemoglobin, 13.7 g/dL; total leukocyte count, 11 000 cells/mm^3^; platelet count, 174 000 cells/mm^3^; and differential counts of neutrophils, 60%, lymphocytes, 30%, and eosinophils, 10%. HIV ELISA was negative. A chest X‐ray showed hyperinflation along with infiltrates on the right lung field (Figure 1B). He was prescribed oral antibiotics (amoxicillin‐clavulanic acid), B_2_ agonist (both oral and nebulization), and steroid (budesonide) nebulization. The child responded well to the treatment, but symptoms reoccurred again after one week. He was then diagnosed as a case of moderate persistent asthma (based on night‐time weekly symptoms, eosinophil count, 10%, and improvement with inhaled B_2_‐agonist along with spirometry findings for asthma). Spirometry showed the FEV_1_/FVC ratio of less than 80% predicted with a post‐bronchodilator improvement response of 12%. The child was started on a meter‐dose inhaler with two‐puff twice‐daily inhaled long‐acting B_2_‐agonist with a steroid (formoterol fumarate 6 mcg and budesonide 200 mcg inhaler). The child was symptom‐free for about 2‐3 months, but symptoms reappeared even on the above medication. At this point, alternate diagnosis rather than recurrent and persistent pneumonia was sought. Further, the chest CT was done, which showed features suggestive of mucus impaction (Figure 2).

**FIGURE 3:**
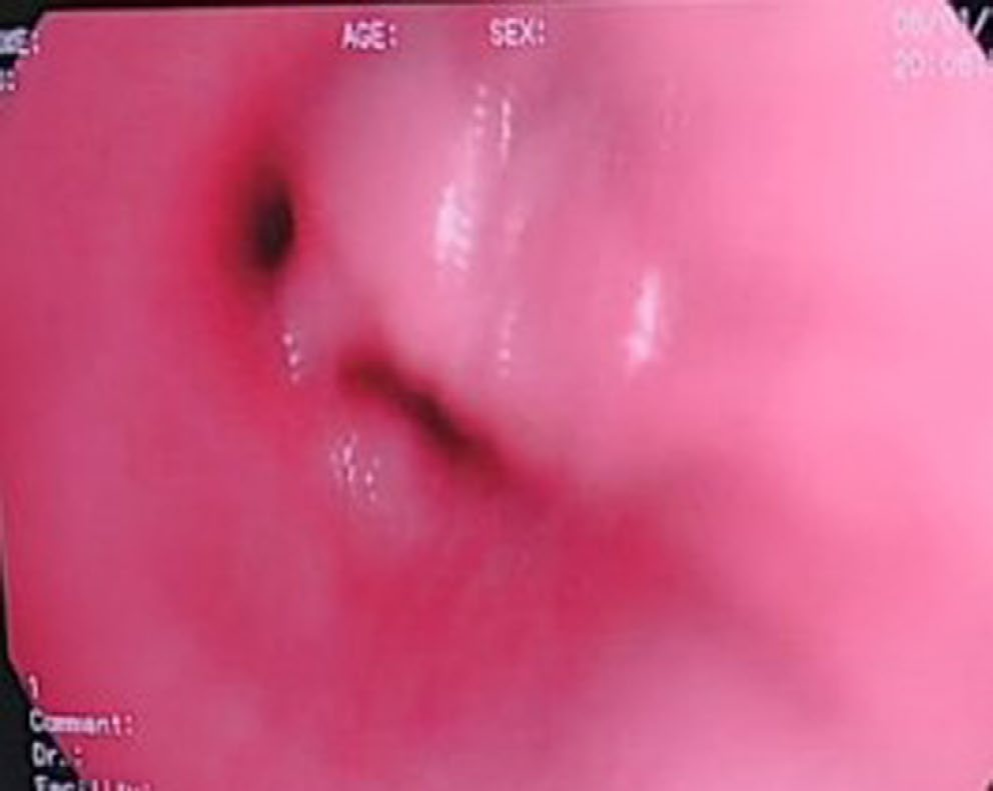
Soft tissue growth at bronchus intermedius

**FIGURE 4:**
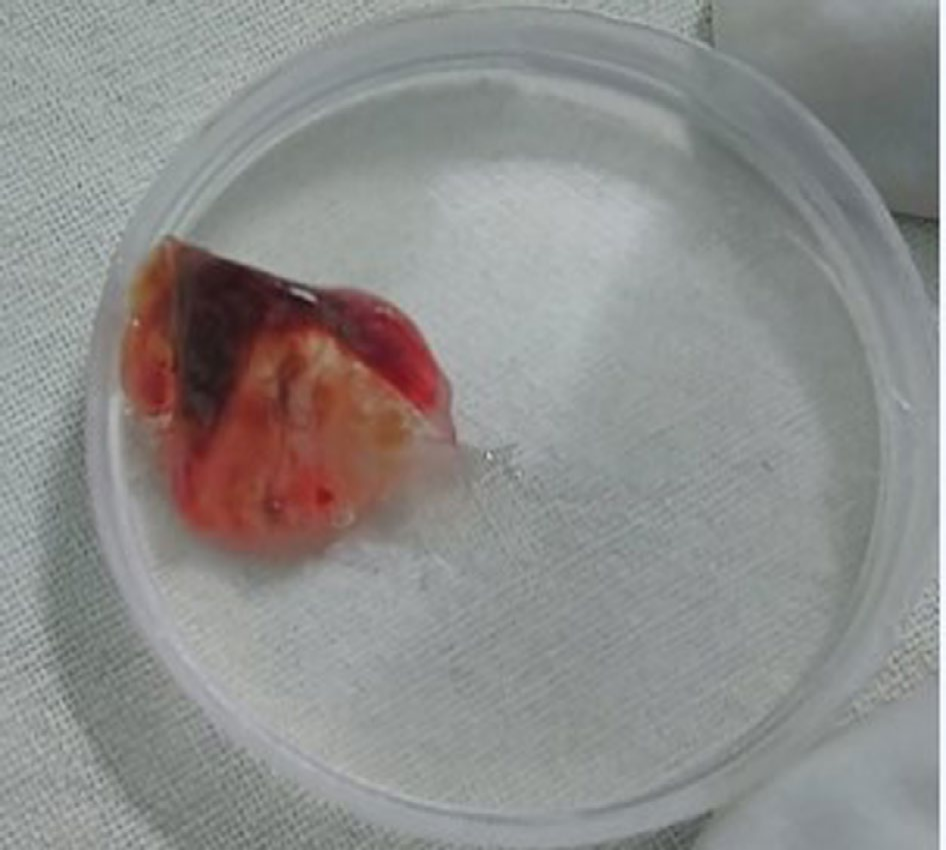
5 mm chicken meat removed from right bronchus intermedius

Flexible bronchoscopy was planned and performed under intravenous sedation, which showed a foreign body (chicken meat) residing on right bronchus intermedius along with soft tissue growth mobile during coughing. The soft tissue growth was seen in the division of bronchus intermedius, partially obliterating both middle and lower lobe bronchus (Figure 3). The chicken meat (size about 5 mm) was removed from the right bronchus intermedius along with granulation tissue (Figure 4). An endobronchial biopsy was obtained and sent for histopathological examination along with the foreign body. Biopsy report showed benign bronchial tissue with a bit of granulation tissue from the endobronchial specimen and bacterial colonies with few epithelial cells from the foreign body.

The child was discharged on oral antibiotics for one week. Follow‐up after one week showed improvement in the respiratory symptoms with normal chest examination findings. The child was again followed up in a three‐month interval where he was found to be asymptomatic with normal examination findings.

## 3 DISCUSSION

Many children with asthma respond well to standard treatment. Periodically, pediatric pulmonologists may encounter asthmatic children who do not improve to the standard treatment regimens even after checking the compliance and the proper drug delivery. They are often labeled as difficult asthma. These children require further investigations, and differentials such as left ventricular failure, vocal cord dysfunction, cystic fibrosis, tumors, chronic upper airway obstruction, and FB aspiration should be considered.^6^, ^7^ FB aspiration can closely mimic bronchial asthma exacerbation. Therefore, a high index of clinical suspicion with the absence of atopy in such patients, acute‐onset disease, unilateral chest examination findings, and failure to respond with bronchodilators can help in the diagnosis of FB aspiration.^8^

The present case report emphasizes that a child, initially misdiagnosed with difficult asthma, improved completely after bronchoscopic removal of the FB from the right bronchus. Although FBs can impact at any site of airways (an inlet of the larynx to terminal bronchioles), however, the right main bronchus remains a common site for lodgement of FB. The main reasons for the impaction of FBs in the right bronchus are likely due to greater diameter and lesser angle of divergence of right bronchus in comparison with the left bronchus and therefore creating a relatively straight pathway from the larynx to the bronchus.^9^

Although tracheobronchial FBs commonly occur in infants and young children, they are not unusual in older children and adults. FB is usually missed in those children when the pediatricians fail to consider FB aspiration as a possibility and do not take an appropriate history. Such children typically visit the pediatrician for their acute or chronic respiratory symptoms. In our case also, the child did not give any such account of aspiration and presented to several pediatric clinics for his cough, respiratory distress, and chest tightness. The presence of the symptoms, as mentioned above, along with wheeze on auscultation, can easily confuse the treating pediatricians to consider the diagnosis of bronchial asthma in the child.

Initial symptoms in a child with FB aspiration are cough, choking, and respiratory distress. As the FB lodges to the distal part, the non‐productive cough, along with wheeze, is important clinical finding. Occasionally, fever, stridor, chest pain, and sternal discomfort can also occur. Children with laryngotracheal FB usually present with cough, stridor, hoarseness, and respiratory distress. The severity of symptoms and presence of clinical signs, however, depend on the site of impaction, degree of obstruction, and whether the FB is fixed or movable.^10^, ^11^, ^12^

FB aspiration generally has three phases. In the initial phase, there is choking, gagging, and paroxysms of coughing or airway obstruction with parents giving a definitive history of this phase only if witnessed. In the latent phase, coughing and protective mechanisms became fatigued and asymptomatic, which can last hours to weeks. Complications occur in the third phase when obstruction, erosion, or infection causes hemoptysis, pneumonia, atelectasis, abscess, or fever. The longer duration of the foreign body in situ produces granulation tissue resulting in small airway lumen, which leads the symptoms to aggravate.^13^

The presence of wheeze in the child can be explained due to the narrowing of the airway lumen caused by chronic inflammation and the formation of granulation tissues. The particular interest in our case was its late presentation (18 months after aspiration), which on bronchoscopic removal showed granulation tissue on histopathological examination. Saquib et al reported that 21.8% of tracheobronchial FB cases in children presented more than one month after aspiration and the delayed diagnosis was attributed to multiple factors (eg, misdiagnosis by treating pediatrician, failure to seek early medical advice, late referral or family members not present during the choking crisis).^14^ In a 10‐year review of 135 children with FB aspiration by Tan et al, 17.7% of children had delayed diagnosis due to physician‐related factors, whereas 15.5% of children presented late as the aspiration event was unwitnessed by the parents.^3^

Chest X‐ray in confirmed FB aspiration can be normal. In a series of 189 children, 47.6% of children had a normal chest X‐ray.^15^ A chest X‐ray in our case showed initial hyperinflation of the right lung field, suggesting that the FB was not radiopaque. The first X‐ray of our case, which was done before visiting our clinic by the local pediatrician, was almost normal due to which the diagnosis of FB was missed. Children with bronchial FB can be radiographically normal, as only 6%‐20% of aspirated FBs are radiopaque.^14^ The sensitivities and specificities of plain chest X‐ray in detecting FB aspiration are found to be 68%‐74% and 45%‐67%, respectively.^16^, ^17^ In such cases, even hyperinflation, atelectasis, and mediastinal shift should arouse the possibility of FB. Lobar atelectasis and collapse of the entire lobe could occur if the obstruction is complete. The obstructive hyperinflation is mainly due to check valve obstruction, which is the most common finding seen in FB aspiration.^18^

Computed tomography (CT) chest helps in a more accurate diagnosis of FB, where X‐rays are inconclusive.^19^, ^20^ In our case, CT chest showed short segment partial cutoff of right lower lobe bronchus with ill‐defined intraluminal soft tissue.

Flexible fiber‐optic bronchoscopy is considered as a gold standard procedure of diagnosis and treatment for FB as it provides direct visualization of airways where the FB is lodged. Flexible bronchoscopy is also safe, cost‐effective, and preferred by many pediatricians as it avoids the need for general anesthesia in comparison with rigid bronchoscopy.^21^ In the present case, flexible bronchoscopy was used for FB removal. The main disadvantage of using flexible bronchoscopy is FB dislodgement.

## 4 CONCLUSION

FB bronchus should be an important differential in a child who has been misdiagnosed as difficult asthma. A high degree of clinical suspicions is required when a child/parent does not give a history of aspiration. Persistence of symptoms in asthma and lack of response to treatment in such cases should always raise a suspicion of an alternative diagnosis.

## CONFLICT OF INTEREST

None declared.

## AUTHOR CONTRIBUTIONS

NC, SS, and OPK: equally involved in drafting, literature search, and writing of the paper.

## ETHICAL APPROVAL

The case study was approved by the hospital administration for consideration to publication.

## CONSENT

Both verbal and written consents were obtained from the parents regarding the publication of the case and photographs.
